# Melioidosis-Series of Seven Cases from Madurai, Tamil Nadu, India

**DOI:** 10.5005/jp-journals-10071-23139

**Published:** 2019-03

**Authors:** Vithiya Ganesan, Raja Sundaramoorthy, Sankar Subramanian

**Affiliations:** 1,2 Department of Microbiology, Velammal Medical College Hospital and Research Institute, Madurai, Tamil Nadu, India; 3 Department of General Medicine, Velammal Medical College Hospital and Research institute, Madurai, Tamil Nadu, India

**Keywords:** Abscess, Diabetes, Meliodosis

## Abstract

We describe a case series of seven culture proven melioidosis patients presenting during 2014 to 2016 in Madurai, south Tamilnadu. Skin, soft tissue, bone and joint infections were common. All of them were middle aged men except one case. All the cases were reported during the monsoon season. Predisposing factors include diabetes and alcoholism. Despite many case reports and studies from South India, melioidosis still remains undiagnosed, hence under reported from many centers. Delayed diagnosis leads way to sepsis and other complications. Awareness about the preventive measures, earlier clinical and laboratory identification and appropriate management of severe sepsis are required to reduce the burden of this disease.

**How to cite this article:**

Ganesan V, Sundaramoorthy R *et al.*, Melioidosis-Series of Seven Cases from Madurai, Tamil Nadu, India. Indian J Crit Care Med 2019;23(3):149-151.

## INTRODUCTION

Melioidosis is an emerging infectious disease of major public health concern in southeast Asia. Many cases have been reported from different regions of India but represent only tip of the iceberg as they are mostly reported from few large medical centers, where identification is possible^1–4^. In this report, we describe a case series of melioidosis patients presented during 2014 to 2016 in Tamil Nadu.

## CASE SERIES

**Fig. 1:**
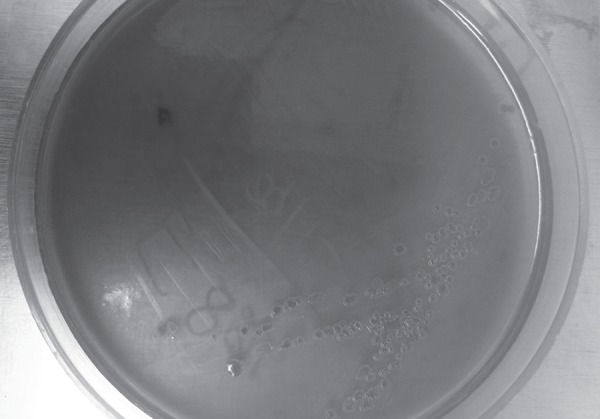
*B. pseudomallei* in MacConkey agar

All of them were middle aged men except one case. They were presented with skin, soft tissue, bone and joint infections. All the cases were reported during the monsoon season. Predisposing factors include diabetes and alcoholism. In all the cases, pus culture grew*Burkholderia pseudomallei*. Gram staining of the pus showed Gram-negative bacilli with bipolar staining. The pus culture showed lactose fermenting pink colonies in MacConkey's agar on 1st day which turned dry and wrinkled on day 2 (Figure 1). Blood agar showed dry and wrinkled colonies on day 2. The organism was confirmed to be*B. pseudomallei* by the above mentioned culture characteristics and standard biochemical methods (positive oxidase and nitrate reduction test, nonfermentingreaction with triple sugar iron agar, hydrolyse arginine, oxidise glucose and lactose). All the isolates were sensitive to cotrimoxazole, doxycycline, ceftazidime, piperacillin tazobactam and meropenem. Bacteremia was confirmed in three cases. Acute renal injury was the most common organ dysfunction found in all the patients. Three patients died of sepsis due to delayed diagnosis and inappropriate management (Table 1).

## DISCUSSON

*B. pseudomallei* is an environmental Gram-negative bacterium and etiological agent of melioidosis. It is generally less virulent in healthy hosts but patients with diabetes mellitus, in particular type 2 diabetes, show a high incidence of melioidosis. In Type 1 diabetes mellitus, use of m-cresol (a preservative) with insulin has an inhibitory effect on the organism^5^. This can be attributed for the high incidence in Type 2 diabetes.

*B. pseudomallei* is known to survive and multiply within cell lines of macrophage/monocyte and neutrophils. Also, the comorbid risk factors for melioidosis contribute by impairing neutrophil function. In diabetes mellitus, neutrophil is structurally and functionally affected thus unable to perform optimally during inflammation. The function still deteriorates during acute and chronic hyperglycemic states. Such type of defects are also observed in association with high alcohol consumption, chronic renal failure and thalassemia. As in tuberculosis, there is a possibility of dormant state of melioidosis in macrophages as there are reported relapses after apparently successful treatment. So, cell mediated immunity plays a prime role in the control of this organism.

**Table 1:** Demographic details, risk factors and outcome of the cases

	*Cases*						
	*1*	*2*	*3*	*4*	*5*	*6*	*7*
Age	52	47	31	65	31	46	67
Sex	M	M	F	M	M	M	M
Presenting month	December	January	January	December	November	September	October
Risk factor	DM	DM	DM	DM, alcoholism	DM	DM, alcoholism	DM, alcoholism
Clinical presentation	Elbow and knee arthritis	Foot cellulitis	Gluteal abscess	Multiple metastatic pyogenic abscess	Osteomyelitis with intramuscular abscess	Pyelonephritis, sepsis, knee arthritis	Cellulitis leg, sepsis
Blood sugar	Recurrent hypoglycemia	116	395	214	216	458	372
Hb	8	12.2	8.2	11.6	10	10.5	9.6
TC	10,900	19,300	12,200	17,200	23,500	13,300	2200
ESR	83	22	55	36	21	33	39
Antibiotic sensitivity	S to CAZ, IMI, CIP, COT, CFS	S to CAZ, COT, DOX	S to CAZ, IMI, MER, CIP, PIT	S to CAZ, MER, IMI, COT, PIT, CIP	S to CAZ, PIT, IMI, CIP, COT	S to CAZ, CIP, IMI, PIT	S to CAZ, CIP, IMI, PIT
Bacteremia	Absent	Absent	Absent	Present	Present	Present	Absent
Organ dysfunction	Acute renal injuy, hypoxic ischemic encephalopathy	Raised renal paramters	Nil	Renal and hepatic dysfunction	Renal dysfunction	Renal and hepatic dysfunction	Acute renal injury
Treatment	MER, CAZ	I & D, CAZ	I & D, PIT	IMI, I & D of inguinal abscess, splenectomy and drainage of liver abscess	I & D of intramuscular abscess	Imipenem for 2 weeks	Imipenem started
Maintenance phase	-	DOX	Amoxyclav	-	-	COT	
Outcome	Died	Recovered	Recovered	Died	Died	Recovered	Lost to followup

CAZ: Ceftazidime, IMI: Imipenem, CIP: Ciprofloxacin, COT: Cotrimoxazole, CFS: Cefoperazone Sulbactum, DOX: Doxycycline, MER: Meropenem, PIT: Piperacillin Tazobactum

*B. pseudomallei* is transmitted by inhalation, ingestion and inoculation. There is a strong association with monsoonal rains, and occupational and recreational exposure to surface water. In our centre, all the cases were reported during the monsoon. Cellulitis, arthritis, osteomyelitis, pyelonephritis and abscesses were the clinical presentations. Skin and soft tissue infections were rapidly progressive, mimicking necrotizing fasciitis from other organisms like *Streptococcus* and filamentous fungi. High proportions of patients can present with internal abscess, like in one of our cases, multiple pyogenic abscess with liver and spleen involvement.

Markers of organ dysfunction including leucopenia, elevated liver enzymes, renal parameters and metabolic derangements (hypoglycemia and acidosis) during admission appear to predict mortality. In the present case series, renal dysfunction and metabolic derangements were markers of impending mortality. As the microbiological clearance is slow, repeated positive cultures and persistent radiological abnormalities does not necessarily mean a poor prognosis.

*B. pseudomallei* exhibits resistance to penicillins, amino- glycosides and relatively insensitive to macrolides and flouroquinolones. So, treatment options are limited. Ceftriaxone and cefotaxime use is associated with a higher failure rate among patients with melioidosis^6^. Ceftazidime and carbapenems remain the drugs of choice during the intensive phase therapy. Use of meropenem especially in severe sepsis is advocated. This is supported by a retrospective study of meropenem use in Australia, in which statistically significant decrease in mortality was seen in meropenem-treated patients with severe sepsis compared with use of ceftazidime only, despite confounding factors like use of Granulocyte colony stimulating factor^7^. Cotrimoxazole with or without doxycycline is used for the prolonged eradication phase. Doxocycline should not be used as monotherapy as drugresistance is expected^8^. Adherence to therapy (24-week course of therapy) is the major factor that prevents relapse.

## CONCLUSION

To diagnose melioidosis promptly, a high index of suspicion in certain clinical settings cannot be overemphasized. Delayed diagnosis leads way to sepsis and other complications. Awareness about the preventive measures, earlier clinical and laboratory identification, and appropriate management of severe sepsis are required to reduce the burden of this disease.
